# Increased B Cell-Activating Factor Expression Is Associated with Postoperative Recurrence of Chronic Rhinosinusitis with Nasal Polyps

**DOI:** 10.1155/2022/7338692

**Published:** 2022-04-08

**Authors:** Fang Zhang, Zhenhang Xu, Xi He, Yi Sun, Chong Zhao, Jianhui Zhang

**Affiliations:** ^1^Department of Otolaryngology, The Third People's Hospital of Chengdu, Chengdu, China; ^2^Department of Otolaryngology, Affiliated Hospital of North Sichuan Medical College, Nanchong, China; ^3^Department of Biomedical Sciences, Creighton University School of Medicine, Omaha, NE, USA

## Abstract

**Background:**

Chronic rhinosinusitis with nasal polyps (CRSwNP) is a common upper airway inflammatory disease with a high postoperative recurrence rate. This study is aimed at exploring the expression of B cell-activating factor (BAFF) in CRSwNP and its association with postoperative recurrence.

**Methods:**

A total of 80 CRSwNP patients, including 40 primary CRSwNP patients and 40 recurrent CRSwNP patients, 40 chronic rhinosinusitis without nasal polyps (CRSsNP) patients, and 40 healthy controls (HC) were enrolled in this study, and the serum and tissue samples were collected. The circulating and tissue BAFF expressions were detected by enzyme-linked immunosorbent assay reverse transcription-polymerase chain reaction and immunohistochemistry. Their clinical values for predicting postoperative recurrence of CRSwNP were evaluated.

**Results:**

We determined serum levels of BAFF were remarkably increased in the CRSwNP group than the CRSsNP and HC groups (*P* < 0.05), and higher concentrations of BAFF were associated with peripheral eosinophil percentage (*r* = 0.614, *P* < 0.001). The serum BAFF concentrations were significantly higher in the recurrent CRSwNP group in comparison with the primary group (*P* < 0.05). Multivariate analysis and receiver operating characteristic (ROC) curve presented that serum BAFF levels were associated with the postoperative recurrence in CRSwNP patients (*P* < 0.05). Moreover, tissue BAFF levels were significantly increased in the CRSwNP group than the HC group, especially in the recurrent CRSwNP group (*P* < 0.05), and enhanced BAFF RNA expressions were correlated with serum BAFF levels (*r* = 0.703, *P* < 0.001).

**Conclusion:**

Our results elucidated that the BAFF expression was enhanced in CRSwNP patients and associated with postoperative recurrence. BAFF could be a serologic biomarker for predicting postoperative recurrence in CRSwNP patients.

## 1. Introduction

Chronic rhinosinusitis (CRS) is a chronic inflammatory disease of the upper respiratory tract which results in clinical syndromes, including nasal discharge, nasal obstruction, and reduction and loss of smell [1, 2]. Previous epidemiologic studies showed that CRS is one of the most common disorders with an estimated prevalence of 2–4% according to the geographic region surveyed [3, 4]. Based on the presence of nasal polyp, CRS is subclassified into CRS with nasal polyps (CRSwNP) and CRS without nasal polyps (CRSsNP), and CRSwNP tends to have severe disease symptoms, poorer prognosis, and a higher rate of postoperative recurrence, because of massive eosinophilic-driven Th2 cytokine infiltration in the nasal mucosa [5–7]. Prior publications revealed that the short-term polyp recurrence rate was approximately 50% 12 months following functional endoscopic sinus surgery, and the long-term recurrence rate was 60%-70% [8–10]. Given that recurrence frequently occurs among CRSwNP patients, it is urgently needed to identify factors associated with postoperative recurrence and explore objective biomarkers for predicting its recurrence, which was pivotal to develop treatment strategies, adjust follow-up protocols, and achieve personalized treatments.

B cell-activating factor (BAFF), also known as B lymphocyte stimulator (BLyS) and tumor necrosis factor superfamily 13B (TNFSF13B), is widely expressed on monocytes, macrophages, dendritic cells, and stromal cells and exhibits a variety of biological functions, including maintaining B cell homeostasis, promoting B cell survival and differentiation, and regulating the function of T cells [11–13]. Prior publications found that the BAFF was overexpressed in local tissue and peripheral blood and closely linked with underlying pathogenesis of malignant tumor [14], autoimmune diseases [15, 16], inflammatory bowel disease [17, 18], and transplantation rejection [19, 20]. Currently, the roles of BAFF in airway inflammatory disease have attracted close attention, and increased levels of BAFF were observed in serum and sputum specimens in allergic asthma patients [21, 22]. Wang et al. [13, 23] presented that B cell-activating factor was crucial to promote B cell survival in ectopic lymphoid tissues in nasal polyps, suggesting that BAFF was involved in the physiopathologic mechanism of CRSwNP and might serve as an objective biomarker for its individualized treatment. Here, this present study is aimed at detecting the BAFF levels in serum and tissue of CRSwNP and assess its potential value in predicting postoperative recurrence.

## 2. Materials and Methods

### 2.1. Patients

We recruited 40 healthy controls, 40 CRSsNP patients, and 80 CRSwNP patients (40 primary CRSwNP and 40 recurrent CRSwNP) treated in our department between January 2021 and July 2021. CRSwNP was diagnosed according to physical examination, nasal endoscopy images, and sinus computed tomography (CT) findings referring to the guidelines of the European Position Paper on Rhinosinusitis and Nasal Polyps 2012 [24]. Atopic status was confirmed by physicians based on disease history, skin tests and/or specific IgE tests, and pulmonary function. All CRSsNP and CRSwNP patients met the diagnostic criteria referring to the guidelines of the European Position Paper on Rhinosinusitis and Nasal Polyps 2012. Recurrent CRSwNP was defined when the presence of typical symptoms despite the rescue regimen of antibiotics and oral steroids during the follow-up period and endoscopic images and/or computed tomography (CT) scan evidence were obtained during outpatient clinic follow-up as previously described [25–27]. We excluded these patients if they had fungal sinusitis, allergic fungal rhinosinusitis, nasal or sinus malignancy; accompanying autoimmune diseases or eosinophilic diseases; consumption of immunotherapy drugs, antibiotics, corticosteroids, or antiallergic drugs 4 weeks before enrollment in this study. Forty age- and sex-matched healthy controls (HCs) without nasal or sinus inflammatory diseases were similarly recruited as control group, and they did not receive any immunotherapy, antibiotics, or corticosteroid therapy within 4 weeks and had no severe heart and kidney dysfunction conditions or inflammatory or autoimmune diseases. This study was approved by the Medical Ethics Committee of the Third People's Hospital of Chengdu. All subjects were asked to sign informed consent before they were included.

### 2.2. Serum BAFF Level Detection

Fasting blood samples were collected in the morning with non-anticoagulant vacuum blood collection tube from all participants and stored at room temperature for 2 hours to clot. The coagulated blood samples were centrifuged, and the supernatants were harvested and stored at -80°C until use. All serum samples were thawed and centrifuged before use, and the serum BAFF levels were quantified by commercial ELISA kit (CUSABIO, Wuhan, China) following the manufacturer's instructions. All assays were conducted by experimenters who were blinded to the diagnosis and clinical manifestations. All samples were tested in duplicate to improve assay precision.

### 2.3. Real-Time Polymerase Chain Reaction Analysis

All tissue specimens were collected during surgery and immediately frozen in liquid nitrogen. Tissue total RNA was extracted with TRIzol reagent (New Cell & Molecular Biotech, Suzhou, China) and reverse transcribed into cDNA using SureScript first strand cDNA synthesis kit (US EVERBRIGHT, Suzhou, China). Primers for BAFF and glyceraldehyde-3-phosphate dehydrogenase (GAPDH) were designed and synthesized by Sangon Biotechnology (Shanghai, China). The real-time polymerase chain reaction (qRT-PCR) was performed using 100 ng of cDNA and SYBR Green qPCR SuperMix (US EVERBRIGHT, Suzhou, China) following the protocols. The mRNA expression of gene was calculated using the comparative threshold cycle (2−*ΔΔ*CT) method. The primers used for amplifying GAPDH and BAFF are displayed in Table S1.

### 2.4. Hematoxylin-Eosin Staining and Immunohistochemistry

Tissue specimens were fixed with 10% formalin, embedded with paraffin, and then sectioned 4 *μ*m thickness and stained with hematoxylin-eosin (HE) staining. Immunohistochemistry analysis (IHC) was conducted as previously described [28, 29], including antigen retrieval, primary antibody to BAFF (Affinity Biosciences, Changzhou, China) and secondary antibody incubation. Streptavidin biotin complex (SABC) kit (Weiao Biological Technology) was utilized for visualization. Histological changes and BAFF expressions in the sections were observed by two independent pathologists, and the images were selected and displayed in each group.

### 2.5. Statistical Analysis

All data were expressed as the mean ± standard deviation. One-way analysis of variance (ANOVA) or Student's *t*-test was conducted when the variables distributed normally; otherwise, Kruskal-Wallis *H* test or Mann–Whitney *U* test was performed. Spearman correlation test was applied to present the associations between BAFF expression and clinical variables. Multivariate analysis and receiver operating characteristic (ROC) curves were performed to evaluate the value of BAFF levels in predicting CRSwNP postoperative recurrence. Statistical analyses were performed with SPSS statistics software version 25.0 (IBM, Chicago, IL, USA). A *P* value < 0.05 was defined as statistically significant.

## 3. Results

### 3.1. Baseline Data of All Subjects

Demographic and clinical data of all participants are displayed in Table 1. The rate of allergic rhinitis, peripheral eosinophil count, and percentage were significantly increased in the CRSwNP group than the CRSsNP and HC groups (all *P* < 0.05). The VAS score and Lund-Mackay score were higher in the CRSwNP group than the CRSsNP group (all *P* < 0.05). No significant differences were observed among three groups for the other variables. As shown in Table 2, the patients in the recurrent group had a significantly higher rate of allergic rhinitis, peripheral eosinophil count and percentage, and VAS score than those in the primary group (all *P* < 0.05), and no statistic difference was observed between two groups for the other variables.

### 3.2. BAFF Levels in Serum and Tissues of CRSwNP

**Figure 1:**
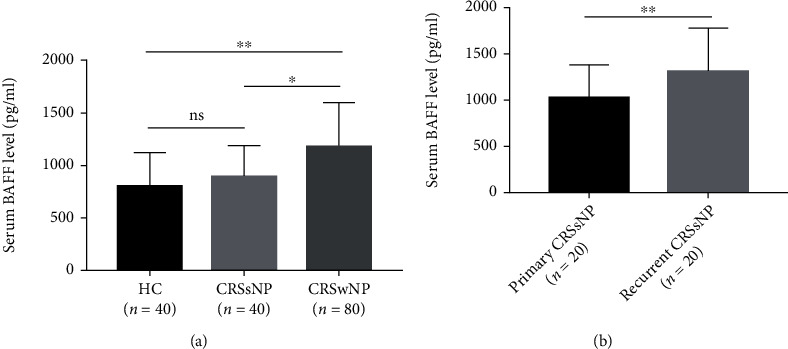
Comparison of serum BAFF levels among three groups. (a) Serum BAFF concentrations were increased in the CRSwNP group than the CRSsNP and HC groups. (b) Serum BAFF concentrations in the recurrent CRSwNP group were higher than the primary CRSwNP group. BAFF: B cell-activating factor; CRSwNP: chronic rhinosinusitis with nasal polyps; CRSsNP: chronic rhinosinusitis without nasal polyps; HC: healthy control; NS: no significance. ^∗^*P* < 0.05 and ^∗∗^*P* < 0.01.

**Figure 2:**
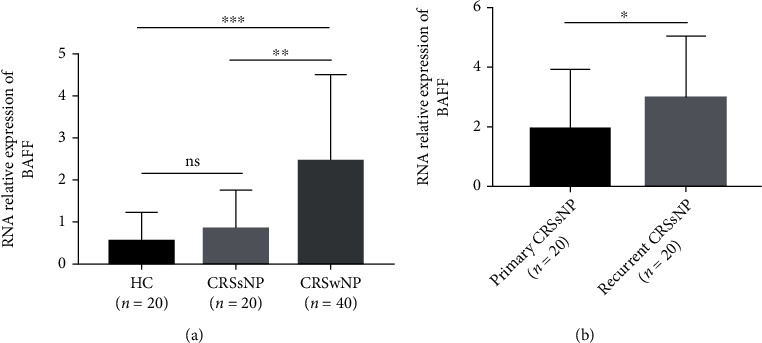
Comparison of tissue BAFF mRNA levels among three groups. (a) Tissue BAFF relative expressions were enhanced in the CRSwNP group than the CRSsNP and HC groups. (b) Tissue BAFF relative expressions were higher in the recurrent CRSwNP group than the primary CRSwNP group. BAFF: B cell-activating factor; CRSwNP: chronic rhinosinusitis with nasal polyps; CRSsNP: chronic rhinosinusitis without nasal polyps; HC: healthy control; NS: no significance. ^∗^*P* < 0.05 and ^∗∗^*P* < 0.01.

**Figure 3:**
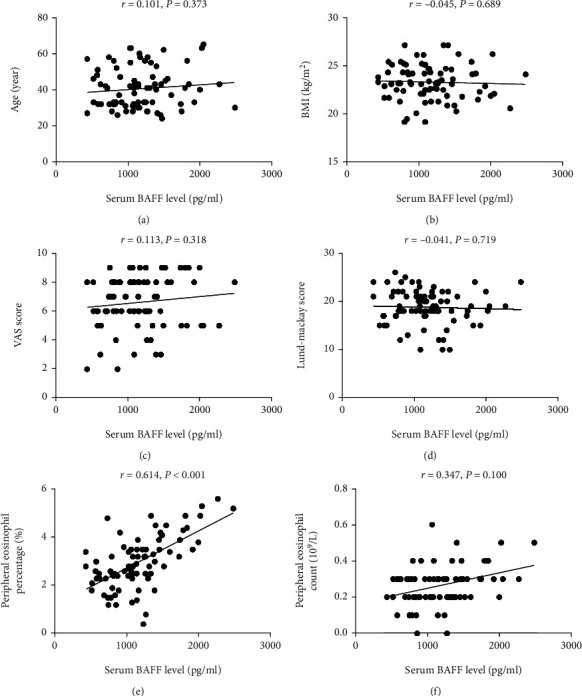
The associations between serum BAFF levels and clinical variables in CRSwNP patients. Elevated serum BAFF levels exhibited positive correlation with peripheral (e) eosinophil count, but did not link with (a) age, (b) BMI, (c) VAS score, (d) Lund-Mackay score, and (f) peripheral eosinophil percentage. BAFF: B cell-activating factor; CRSwNP: chronic rhinosinusitis with nasal polyps; BMI: body mass index; VAS: visual analog scale.

**Figure 4:**
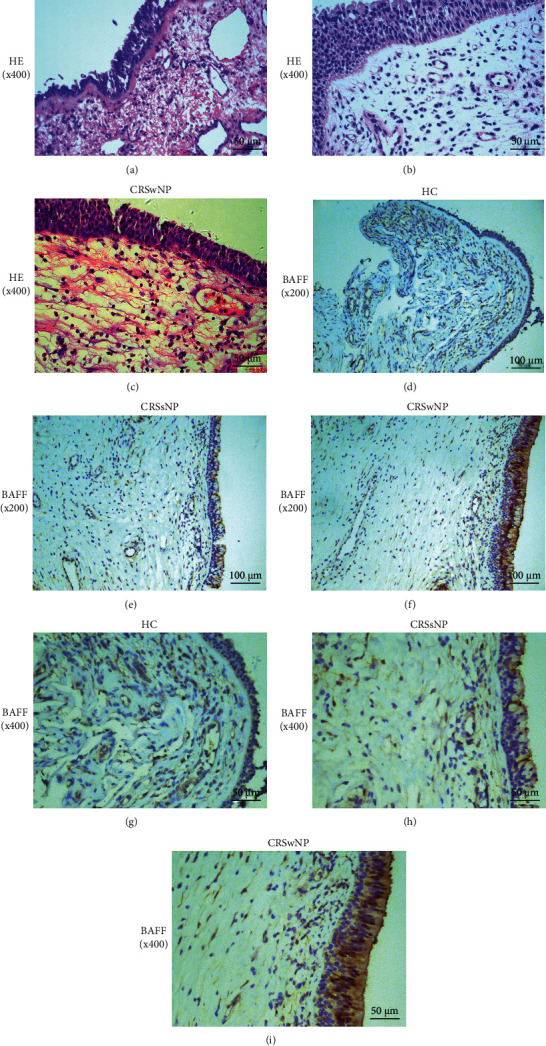
Histopathology and BAFF protein expression in the tissue among three groups. (a–c) Representative H&E images from HC, CRSsNP, and CRSwNP. (d–i) Representative IHC staining of BAFF in HC, CRSsNP, and CRSwNP (magnification, 200x and 400x). BAFF: B cell-activating factor; H&E: hematoxylin and eosin; CRSwNP: chronic rhinosinusitis with nasal polyps; CRSsNP: chronic rhinosinusitis without nasal polyps; HC: healthy control; IHC: immunohistochemistry.

**Figure 5:**
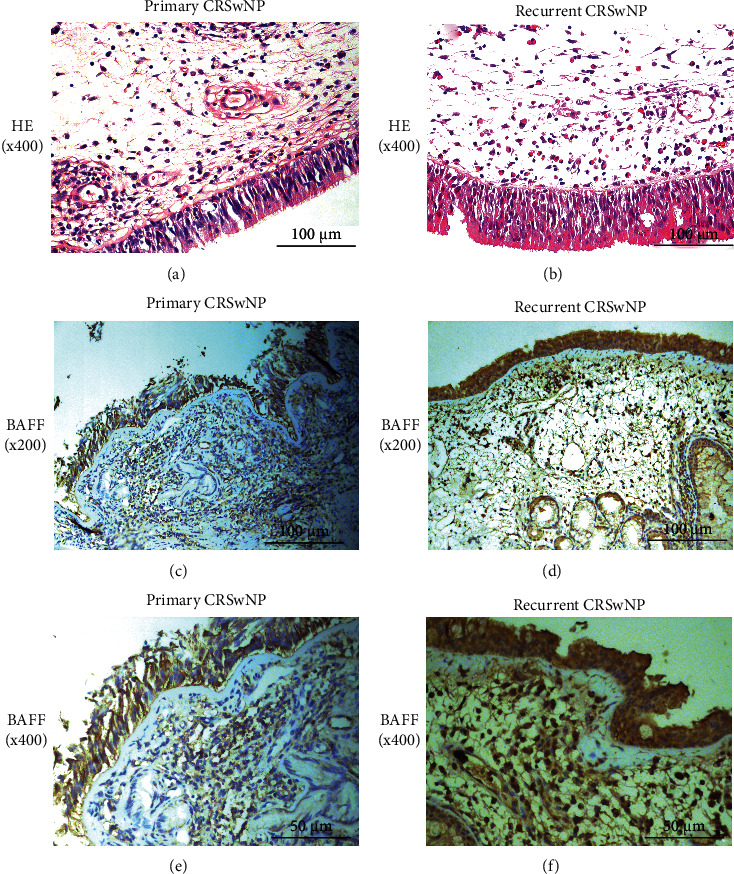
Histopathology and BAFF protein expression in primary and recurrent CRSwNP patients. Representative H&E images from (a) primary and (b) recurrent CRSwNP patients. (c–f) Representative IHC staining of BAFF between two groups (magnification, 200x and 400x). BAFF: B cell-activating factor; H&E: hematoxylin and eosin; CRSwNP: chronic rhinosinusitis with nasal polyps; IHC: immunohistochemistry.

As displayed in Figure 1, the serum BAFF concentrations were 1166.7 ± 433.3 pg/mL in the CRSwNP group, which were markedly higher than those in the CRSsNP group (890.8 ± 292.4 pg/mL, *P* < 0.005) and the HC group (799.1 ± 313.0 pg/ml, *P* < 0.05), but no statistic difference was seen between the CRSsNP and HC groups (*P* < 0.05). The serum BAFF levels were significantly elevated in the recurrent group in comparison with the primary group (1303.7 ± 470.7 pg/mL vs. 1029.7 ± 346.8 pg/mL, *P* < 0.05). Similarly, the BAFF levels were significantly higher in the CRSwNP group than the CRSsNP (*P* < 0.05) and HC (*P* < 0.05) groups, and mRNA expressions were markedly enhanced in the recurrent group in comparison with the primary group (*P* = 0.039, Figure 2). The Spearman correlation results in Table 3 and Figure 3 presented that the elevated serum BAFF exhibited a positive association with peripheral eosinophil percentage (*r* = 0.614, *P* < 0.001) and tissue BAFF RNA expression level (*r* = 0.703, *P* < 0.001). As indicated by representative HE and IHC images in Figures 4 and 5, tissue edema, mucosal tissue remodeling, and inflammatory cell infiltration were observed in CRSwNP and CRSsNP patients, especially in recurrent CRSwNP patients. Strong BAFF immunoreactivity was observed in polyp tissues of CRSwNP patients, but not in CRSsNP patients and HCs. BAFF expressions were markedly enhanced in recurrent CRSwNP patients than in primary patients, especially in nasal epithelial cells and submucosal and glandular cells.

**Figure 6:**
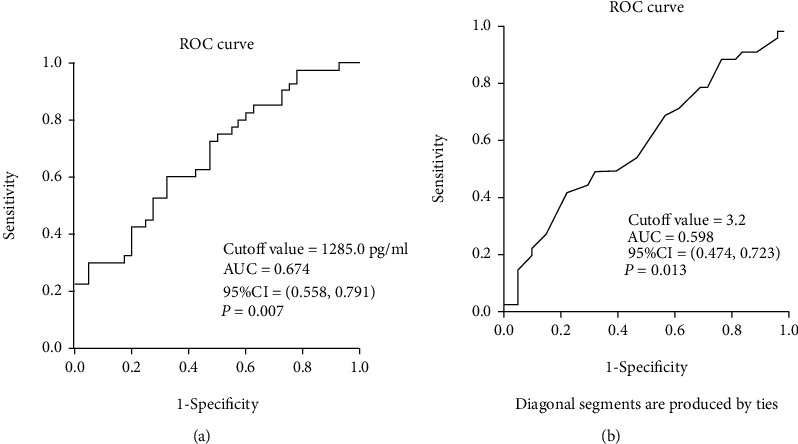
ROC curve and parameters for predicting CRSwNP recurrence: (a) serum BAFF; (b) peripheral eosinophil count. ROC: receiver operating characteristic; CRSwNP: chronic rhinosinusitis with nasal polyps; BAFF: B cell-activating factor.

Identification of the predictive factors associated with CRSwNP recurrence: in order to explore the potential factors associated with CRSwNP recurrence, the variables with significant differences were included in binary logistic regression analysis. The results demonstrated that peripheral eosinophil percentage (OR = 2.012, *P* = 0.026) and serum BAFF level (OR = 1.856, *P* = 0.008) were associated with CRSwNP postoperative recurrence (Table 4). ROC curves in Figure 6 showed that serum BAFF (AUC = 0.747) presented a better potential value in predicting CRSwNP postoperative recurrence than peripheral eosinophil percentage (AUC = 0.681).

## 4. Discussion

In the current study, we demonstrated that soluble form and membrane-bound form of BAFF were elevated in CRSwNP patients, and both forms of BAFF were markedly enhanced in recurrent CRSwNP patients in comparison with primary CRSwNP patients. Statistical analysis results showed that serum BAFF exhibited potential predictive value for CRSwNP recurrence. Taken together, these results suggested that BAFF expression contributed to the CRSwNP recurrence and serum BAFF might serve as a potential biomarker for predicting postoperative recurrence in CRSwNP patients.

Currently, an increasing body of evidence suggested that B cell biology and function were deeply involved in the pathogenesis of upper airway diseases including allergic rhinitis and CRS [30, 31]. Exaggerated or dysregulated B cell function can drive T2 mucosal inflammatory states in nasal mucosa via local antibody production and other inflammatory cell recruitment [32]. BAFF is expressed on several immune and nonimmune cells and acts as a crucial biomolecule in sustaining B cell homeostasis [16, 33]. Previous studies found that BAFF overexpressed peripheral blood and local tissues in tumor, inflammatory, and autoimmune diseases, and soluble form of BAFF was demonstrated to be a promising biomarker in reflecting disease activity and predicting prognosis [14, 17, 21, 33]. A recent study reported that both soluble and tissue BAFF levels were upregulated in renal transplant recipients, and serum BAFF conferred a potential value in distinguishing chronic antibody-mediated rejection patients from stable kidney transplant patients [19]. Alturaiki et al. showed that BAFF expressions were elevated in allergic asthma patients; enhanced concentrations of BAFF activated BAFF receptor signaling pathway on B cells and promoted IgE production and then aggravated inflammation response in the airways [22, 34]. In the present study, we observed that both circulating and tissue BAFF levels were elevated in the CRSwNP patients in comparison with HCs and serum BAFF levels associated with peripheral blood eosinophilia. Interestingly, no statistic difference was seen in BAFF expression between CRSsNP patients and HCs. Accordingly, CRSwNP was characterized by Th2 immune response and eosinophil recruitment, while Th1 immune response was predominated in the pathogenesis of CRSsNP [35–37]. Therefore, we speculated that stimuli such as bacteria, allergen, and virus could promote the BAFF production and activate B cell differentiation and antibody accumulation and then trigged Th2 immune response and eosinophil recruitment, resulting in excessive inflammatory states in CRSwNP.

Although medical treatment and nasal endoscopic surgery were proven to be effective in improving the quality of life and clinical outcomes in CRSwNP patients, CRSwNP still had a high rate of recurrence, especially in those patients with tissue eosinophilia [38, 39]. Therefore, exploring risk factors associated with CRSwNP recurrence and predicting its recurrence are extremely challenging and urgently needed for rhinologists. Although several variables and indicators were reported to be correlated with postoperative recurrence, including accompanied asthma [10], Charcot-Leyden crystal [40] and peripheral parameters [41], and activated leukocyte cell adhesion molecule [42], there was no reliable biomarker available in clinical practice. Here, we firstly found that BAFF expressions were significantly enhanced in recurrent CRSwNP patients, and serum levels were closely linked with the rate of CRSwNP postoperative recurrence. Statistical analysis results demonstrated that serum BAFF was a promising value for predicting CRSwNP recurrence. Previous publications showed that the Th2 inflammation response and eosinophil infiltration were recognized as major factors associated with poorly controlled disease and recurrence in CRSwNP [35, 43, 44]. In this study, we found that peripheral eosinophil count was increased in CRSwNP patients and associated with postoperative recurrence, which was in accordance with prior studies. We also found that serum BAFF levels positively correlated with peripheral eosinophil count, which suggested that BAFF might promote eosinophilia and be emerged in recurrence. A recent study revealed that elevated circulating BAFF levels could promote Th2 inflammation and eosinophilic inflammation in allergic asthma [34]. The high concentrations of circulating BAFF would drive B-dependent Th2 activation and cytokine secretion and promote the eosinophil infiltration into the tissue and then increase the risk of postoperative recurrence in CRSwNP patients. All above data suggested that serum BAFF might serve as a novel biomarker for preoperatively predicting recurrence in CRSwNP patients.

Several limitations should be stated in this study. First, the sample size in this study was relatively small, making the statistical analysis challenging. Second, all participants were recruited in a single medical center with the same ethnic background, which increased the risk of selection and limited its generalization. Lastly, there is no internationally consensual diagnostic criterion for recurrent CRSwNP. Further multicenter studies with larger sample size and unified diagnostic criteria are needed to confirm the results of this study.

In summary, our study confirmed the association between BAFFM and CRSwNP. We firstly demonstrated that BAFF was overproduced in CRSwNP and associated with postoperative recurrence. BAFF could be a serologic biomarker for predicting postoperative recurrence in CRSwNP patients and providing a novel intervention target to improve precise treatment.

## Figures and Tables

**Table 1:** The demographic and clinical characteristics among three groups.

Variables	HC (*n* = 40)	CRSsNP (*n* = 40)	CRSwNP (*n* = 80)	*P* value
Age (years)	37.0 ± 7.2	38.6 ± 7.9	40.1 ± 10.8	0.652
Gender (male/female)	22/18	26/14	47/33	0.652
Smoker (yes/no)	10/30	12/28	23/57	0.870
BMI (kg/m^2^)	22.6 ± 1.7	22.6 ± 1.7	23.3 ± 1.8	0.302
Allergic rhinitis (yes/no)	0/40	6/34	18/62	0.005
Asthma (yes/no)	0/40	3/37	10/70	0.061
Peripheral eosinophil count (10^9^/L)	0.1 ± 0.1	0.2 ± 0.1	0.3 ± 0.2	<0.001
Peripheral eosinophil percentage (%)	2.0 ± 0.8	2.3 ± 1.1	3.0 ± 1.0	<0.001
VAS score	—	5.4 ± 1.6	6.6 ± 1.8	0.002
Lund-Mackay score	—	16.4 ± 3.1	18.8 ± 3.7	0.002

HC: healthy control; CRSsNP: chronic rhinosinusitis without nasal polyps; CRSwNP: chronic rhinosinusitis with nasal polyps; BMI: body mass index; VAS: visual analogue scale.

**Table 2:** The demographic and clinical parameters between primary and recurrent CRSwNP group.

Variables	Primary CRSwNP group (*n* = 40)	Recurrent CRSwNP group (*n* = 40)	*P* value
Age (years)	40.7 ± 10.4	39.5 ± 11.2	0.614
Gender (male/female)	25/15	22/18	0.650
Smoker (yes/no)	10/30	13/27	0.622
BMI (kg/m^2^)	23.4 ± 1.8	23.2 ± 1.8	0.749
Allergic rhinitis (yes/no)	4/36	14/26	0.014
Asthma (yes/no)	3/37	7/33	0.311
Peripheral eosinophil count (10^9^/L)	0.2 ± 0.1	0.3 ± 0.1	0.004
Peripheral eosinophil percentage (%)	2.6 ± 1.0	3.3 ± 1.0	0.005
VAS score	6.2 ± 1.9	7.0 ± 1.5	0.044
Lund-Mackay score	18.0 ± 4.0	19.1 ± 3.2	0.461

CRSwNP: chronic rhinosinusitis with nasal polyps; BMI: body mass index; VAS: visual analogue scale.

**Table 3:** Association between BAFF expression and clinical variables in CRSwNP patients.

Variable	Serum BAFF level		BAFF RNA expression level	
	*r*	*P* value	*r*	*P* value
Age	0.101	0.373	0.116	0.296
BMI	-0.045	0.689	-0.201	0.512
VAS score	0.113	0.318	0.278	0.310
Lund-Mackay score	-0.041	0.719	0.476	0.053
Peripheral eosinophil	0.614	<0.001	0.317	0.175
Percentage (%)				
Peripheral eosinophil count (10^9^/L)	0.347	0.010	0.296	0.347
BAFF mRNA expression level	0.703	<0.001	—	—
Serum BAFF level	—	—	0.703	<0.001

BAFF: B cell-activating factor; CRSwNP: chronic rhinosinusitis with nasal polyps; BMI: body mass index; VAS: visual analog scale.

**Table 4:** Multivariate analysis of factors associated with the recurrence of CRSwNP.

Variables	OR	95% CI	*P*
Allergic rhinitis (yes/no)	1.294	0.730-2.137	0.258
Peripheral eosinophil count (10^9^/L)	1.631	0.802-1.869	0.313
Peripheral eosinophil percentage (%)	2.012	1.238-3.148	0.026
Serum BAFF level (ng/mL)	1.856	1.173-4.267	0.008

CRSwNP: chronic rhinosinusitis with nasal polyps; BAFF: B cell-activating factor; OR: odds rate; CI: confidence interval.

## Data Availability

The raw data supporting the conclusions of this article will be made available by the authors, without undue reservation.

## Supplementary Materials

Supplementary Materials Table S1 shows the primer sequences for qRT-PCR.
