# Supplementary material for: Increased B Cell-Activating Factor Expression Is Associated with Postoperative Recurrence of Chronic Rhinosinusitis with Nasal Polyps
Source: Mediators Inflamm. 2022 Apr 8;2022:7338692. doi: 10.1155/2022/7338692 (PMC9012647; doi:10.1155/2022/7338692)
Supplement: Supplementary Materials — Table S1 shows the primer sequences for qRT-PCR. [file 7338692.f1.docx]

Table S1 Primer sequences

| Gene | Primers |
| --- | --- |
| GAPDH | Forward: 5’-CTCCTCCTGTTCGACAGTCAGC-3’ |
|  | Reverse: 5’-CCCAATACGACCAAATCCGTT-3’ |
| BAFF | Forward: 5’-GAAGCGATAAGTGGAGTCAGTTTCA-3’ |
|  | Reverse: 5’-CCACATTAGCAGCAACACCAGA-3’ |
